# Overexpression of CD86 enhances the ability of THP‐1 macrophages to defend against *Talaromyces marneffei*

**DOI:** 10.1002/iid3.740

**Published:** 2022-11-18

**Authors:** Rifeng Chen, Di Yang, Linxia Shen, Jinling Fang, Raqib Khan, Donghua Liu

**Affiliations:** ^1^ Department of Dermatology The First Affiliated Hospital of Guangxi Medical University Nanning People's Republic of China

**Keywords:** CD86, lentivirus, overexpression, *Talaromyces marneffei*, THP‐1 macrophages

## Abstract

**Background:**

Macrophages are the first line of defense against *Talaromyces marneffei*. CD86 is a surface molecule expressed on antigen‐presenting cells, such as macrophages, that provide costimulatory signals necessary for T cell activation and survival. In a prior study, it was shown that as infection progressed, CD86 expression levels in macrophages considerably declined while CD86 concentrations in the supernatant significantly increased. Additionally, M1 macrophage polarization was insufficient and switched to M2 macrophage polarization. Besides costimulation, however, additional roles of CD86 are not known or well‐studies. Therefore, we hypothesized that upregulating CD86 on macrophages might promote *T. marneffei* defense.

**Methods:**

A lentivirus vector, called Lenti‐CD86, was used to infect THP‐1 cells to overexpress secretory CD86. Through killing assay, nitric oxide detection, and cytokine detection, the capacity of THP‐1 macrophages to phagocytose and kill *T. marneffei* was examined.

**Results:**

In the current study, Lenti‐CD86 transfection of THP‐1 cells resulted in a signifant expression of CD86. Additionally, the THP‐1 macrophages stably transfected with Lenti‐CD86 showed higher nitric oxide and IL‐1β production, faster polarization, and stronger phagocytosis and killing capabilities than the non‐transfected or control virus transfected cells.

**Conclusion:**

Our study shows that lentivirus‐mediated CD86 overexpression improves THP‐1 macrophages' capacity to phagocytose and eliminate *T. marneffei*.

## Graphical abstract

In the current study, Lenti‐CD86 transfection of THP‐1 cells significantly boosted CD86 synthesis. Additionally, the THP‐1 macrophages stably transfected with Lenti‐CD86 have higher nitric oxide and IL‐1β production, faster polarization, and stronger phagocytosis and killing capabilities than the non‐transfected or control virus transfected cells.

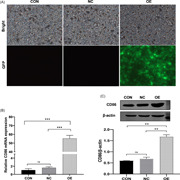

## 1 INTRODUCTION

*Talaromyces marneffei* is an opportunistic pathogenic fungus. Human infection depends on the virulence of the pathogen and the host's immune response. The host's immune response is more important in *T. marneffei* infection than other fungi because *T. marneffei* usually infects an immunodeficient or immunosuppressed host. When *T. marneffei* enters the human body, it remains in macrophages in the form of fission yeast, escapes oxidative killing of host cells and causes immunosuppression. If macrophages are unable to phagocytize and kill the invading *T. marneffei* spores promptly, *T. marneffei* will multiply rapidly in the body.^1^ Therefore, macrophages are the first line of defense against *T. marneffei*.

The killing mechanism of macrophages toward *T. marneffei* involves destroying the fungal pathogens by inducing an unfavorable intracellular environment, such as oxidation and acidification of phagocytes, a lack of intracellular nutrition, the activation of hydrolytic enzymes, the activation of adaptive immune responses, and so forth. All these factors affect the normal metabolism of the pathogen and result in killing the pathogen.^2^ CD86 is usually expressed on the surface of antigen‐presenting cells such as macrophages, B lymphocytes, and dendritic cells; and is involved in the pathogenesis of inflammation. As the second signal of T lymphocyte activation, CD86 promotes the activation, proliferation, and function of T lymphocytes.^3^ Many pathogens can affect the costimulatory pathway mediated by CD28‐CD80/CD86 or the expression of CD80/CD86 on antigen‐presenting cells. For example, superantigens secreted by *Staphylococcus aureus* and *Streptococcus pyogenes* can bind to the homodimer interface of these costimulatory receptors and greatly enhance the binding of CD86 to CD28, resulting in excessive proinflammatory signal transduction.^4^ The Nef protein of HIV‐1 can induce the loss of costimulatory molecules CD80 and CD86 on the surface of antigen‐presenting cells to promote immune escape.^5^ CD86 is also the main costimulatory molecule involved in the primary B lymphocyte response and signal transduction in humoral immunity.^6^ When CD86 is siRNA silenced in B lymphocytes and dendritic cells, it significantly inhibits antigen‐specific allergic reactions and release of IL‐4 and IL‐5.^7^, ^8^ *Bacteroides fragilis* alleviates the symptoms of lupus nephritis by regulating the expression of CD1d and CD86 in B lymphocytes.^9^ The role of the costimulatory molecule CD86 in macrophage phagocytosis and the killing of *T. marneffei* is unknown, despite the fact that it is known that the ability of macrophages to present antigen is crucial during host killing of *T. marneffei*.

In our earlier study, we discovered that *T. marneffei* yeast cells were CD86 stain positive in the cutaneous lesions of patients. Repeated immunohistochemical labeling of *T. marneffei* yeast cells with CD86, CD163, and CD1a revealed that CD86 expression was frequent rather than accidental in both HIV‐negative and HIV‐positive patients.^10^ Furthermore, another study showed that *T. marneffei* did not express CD86 when cultured alone at 37°C, but it did express CD86 when incubated with macrophages.^11^ The level of CD86 expression in macrophages considerably dropped as infection progressed in a time‐dependent manner, while the amount of CD86 in the supernatant significantly increased. Additionally, polarization of the M1 macrophages was insufficient and they polarized to M2 macrophages.^11^, ^12^ To withstand the lethal impact of macrophages, *T. marneffei* may adsorb or take up CD86 in the supernatant produced by them when they come into contact with THP‐1 cells. Therefore, we hypothesized that increasing CD86 on macrophages might enhance immunity against *T. marneffei*. To further investigate the mechanism of interaction between macrophages and *T. marneffei* and discover a new method to combat *T. marneffei* infection, we transfected THP‐1 cells with a CD86 overexpression lentivirus vector plasmid and monitored their coculture with *T. marneffei* conidia.

## 2 MATERIALS AND METHODS

### 2.1 *T. marneffei* strain

*T. marneffei* was previously isolated from the skin lesion of a patient with talaromycosis and the strain was maintained in our laboratory. *T. marneffei* was grown on potato dextrose agar (PDA) at 25°C for 7−10 days. *T. marneffei* conidia were scraped by a sterile loop and 10 ml of sterile phosphate buffer saline (PBS) was added to suspend the collected conidia. The conidia were filtrated three times through eight layers of aseptic gauze. The conidial suspension concentration was adjusted according to the hemocytometer count results.

### 2.2 Cell culture

The THP‐1 cell line used in this study was purchased from the Cell Bank of the Chinese Academy of Sciences. THP‐1 cells were cultured in Roswell Park Memorial Institute medium (RPMI 1640; Gibco; Thermo Fisher Scientific) for subculture containing 10% of heat‐inactivated fetal bovine serum (Gibco; Thermo Fisher Scientific) and 1% penicillin‐streptomycin solution (100 U/ml penicillin G, 0.1 mg/ml streptomycin sulfate; Solarbio). Cell cultures were maintained in an incubator with 5% CO_2_ at 37°C.

### 2.3 Lentivirus transfection

Lentiviruses for overexpressing CD86 (LV‐CD86) and the lentivirus for negative control (LV‐NC) were purchased from Genechem Corporation. Lentivirus LV‐CD86 was a GFP‐CD86 chimeric gene overexpressing virus, and lentivirus LV‐NC was a negative control virus with an empty vector. Twelve‐well plates with THP‐1 cell seeds were continuously transfected for 72 h with either LV‐CD86 or LV‐NC. Puromycin (2 μg/ml) was added. Then stable cell lines were examined for 3 weeks. Cells were also extracted to assess the effectiveness of CD86 overexpression using western blot analysis and quantitative real‐time reverse transcription PCR (qRT‐PCR). The positive control lentivirus transfected cells were referred to as OE cells. The negative transfected cells were designated NC. The cells were given the designation “CON” without any further processing.

### 2.4 *T. marneffei* cocultivation

THP‐1 cells were differentiated into macrophages by incubating with 50 ng/μl PMA (phorbol 12‐myristate 13‐acetate; Meilunbio) for 48 h in Dulbecco s Modified Eagle Medium (DMEM, Gibco; Thermo Fisher Scientific) which contained 10% of heat‐inactivated fetal bovine serum and 1% penicillin‐streptomycin solution. At this point, *T. marneffei* infected macrophages at a multiplicity of infection (MOI) of 10 at 37°C for 24, 48, and 72 h, respectively. The cells and supernatant were collected for further analysis.

### 2.5 Macrophage killing assay

Twenty‐four hours after the coculture, the well plates were washed three times with PBS to elute the *T. marneffei* conidia that were not engulfed by the cells, and the remaining cells were placed in a −80°C refrigerator for 5 min and transferred to a 37°C incubator for heating to release the intracellular phagocytic conidia. The harvested conidia were diluted by adding the same amount of PBS buffer at a ratio of 1:100, and 100 µl of the diluted conidia were plated on YPD flat dishes and the number of colonies was calculated after inverting to incubate in a 27°C incubator for 72 h.

### 2.6 qRT‐PCR analysis

Total RNA was extracted from cells using the RNA prep Pure Cell Kit (Tiangen) and converted to cDNA by using a Prime Script TM RT Reagent kit (Tiangen). qRT‐PCR was performed on a Q5 Gradient Real‐Time PCR Detection System (Bio‐Rad) with the SYBR Green System (SYBR qPCR SuperMix Plus; TIANGEN). The sequences of the primers (Sangon Biotech) are listed in Table 1.

**Table 1:** Primer list

Gene symbol	Primers sequence
GAPDH	F: TGACTTCAACAGCGACACCCA
	R: CACCCTGTTGCTGTAGCCAAA
CD86	F: TTGATTCGGACAGTTGGAC
	R: GTTCTGGGTAACCGTGTAT
IL‐1β	F: GCCAGTGAAATGATGGCTTATT
	R: AGGAGCACTTCATCTGTTTAGG
TNF‐α	F: TGGCGTGGAGCTGAGAGATAACC
	R: CGATGCGGCTGATGGTGTGG
γ‐IFN	F: GAGATGACTTCGAAAAGCTGAC
	R: CCTTTTTCGCTTCCCTGTTTTA

### 2.7 Western blot analysis

RIPA (Cell Signaling) (an abbreviation list is placed in Table 2) was used to lyse cellular proteins, and after adding sodium dodecyl sulfate (SDS) buffers, the mixture was heated for 5 min. Proteins were run on an SDS‐PAGE gel, then transferred to a PVDF membrane and incubated with nonspecific antibodies using 5% skim milk. Each PVDF membrane was subjected to antibody labeling. The antibodies were primary antibody (Rabbit anti‐CD86 antibody from Abcam), β‐actin antibody (Rabbit polyclonal to beta‐actin from Abcam), and secondary antibody (IRDye® 800CW Goat anti‐Rabbit IgG (H + L) from LI‐COR). Finally, the protein bands were photographed by chemiluminescence and calculated quantitatively by ImageJ.

**Table 2:** Abbreviation

Abbreviation	full name
*T. marneffei*	*Talaromyces marneffei*
DMEM	Dulbecco's Modified Eagle medium
NO	nitric oxide
HIV	human immunodeficiency virus
MOI	multiplicity of infection
PMA	phorbol myristate acetate
PBS	phosphate buffer saline
TNF‐α	tumor necrosis factor‐α
IL‐1β	interleukin‐1β
IFN‐γ	interferon‐γ
PDA	potato dextrose agar medium
YPD	yeast extract peptone dextrose medium
COX‐2	cyclooxygenase‐2
iNOS	inducible nitric oxide synthase
APC	antigen presenting cells
CTL‐A4	cytotoxic T‐lymphocyte antigen 4
MAPK	mitogen activated protein kinase
NK	natural killer
RIPA	radioimmunoprecipitation assay

### 2.8 Enzyme‐linked immunosorbent assay (ELISA)

The concentrations of IL‐1β, TNF‐α, and γ‐IFN in cell supernatants collected at 24, 48, and 72 h were tested using commercial ELISA kits (IL‐1β:bsk11001, TNF‐α:bsk11014, γ‐IFN:bsk11013) according to the manufacturer's protocols (Bioss).

### 2.9 Determination of nitric oxide (NO)

The concentrations of NO in cell supernatants collected at 24, 48, and 72 h were tested by using a nitric oxide assay kit according to the manufacturer's protocols (Microwell plate method) (Nanjing Jiancheng). Different reagents were added according to the manufacturer's instructions. The absorbance was then measured at 550 nm with a micro plate reader (SpectraMax13; Molecular Devices).

### 2.10 Statistical analysis

Statistical software, SPSS 22.0, was used for the analysis. The mean ± standard deviation (mean ± SD) was used to represent the data. Student's *t*‐test was used to compare the difference between the two groups. One‐way analysis of variance was used to compare the differences among three or more groups. *p* < .05 was considered statistically significant.

## 3 RESULTS

### 3.1 Lentivirus‐mediated CD86 overexpression in THP‐1 cells

**Figure 1:**
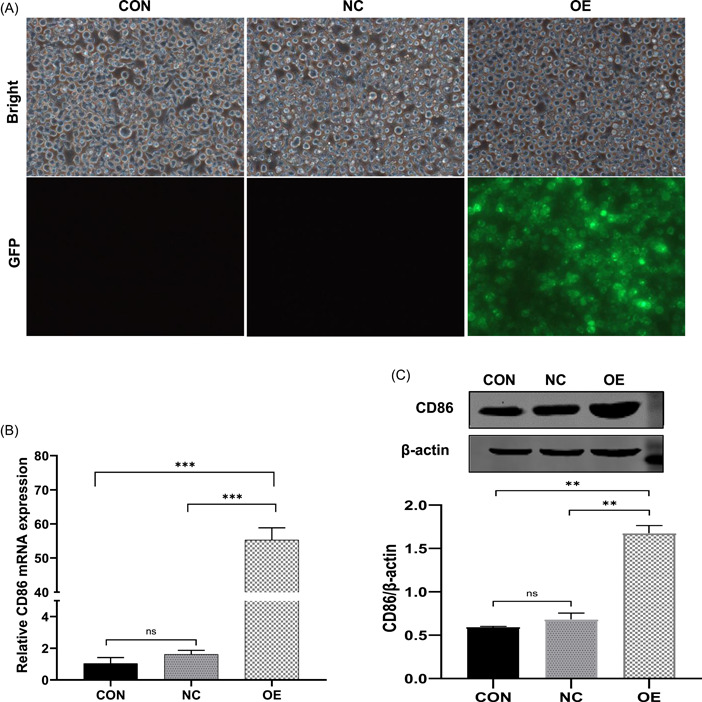
Efficiency of lentivirus‐mediated CD86 overexpression cells. (A) GFP expression was observed in the infected THP‐1 cells by a fluorescence microscopy at 72 h after infection (×200). (B) CD86 expression was clearly increased at the RNA level by LV‐mediated overexpression in THP‐1 cells. (C) Results of western blot revealed the increased CD86 expression in OE group. Data are means ± SD. **p < .01, ***p < .001, ^ns^ *p* > .05. SD, standard deviation.

As shown in Figure 1A, most THP‐1 cells showed GFP‐positive signals at 72 h after transfection with LV‐CD86, indicating that the lentiviruses infected CD86 with high efficiency (Figure 1A). The qRT‐PCR results indicated that there was an overexpression efficiency of approximately 34.21 in THP‐1 cells transfected with LV‐CD86 (rather than LV‐NC) (Figure 1B). Similarly, overexpression efficiency was further determined in THP‐1 cells transfected with LV‐CD86 or LV‐NC via western blot analysis. The western blot analysis results suggested that LV‐CD86 efficiently increased CD86 expression in THP‐1 cells at the protein level (Figure 1C). LV‐NC transfection showed no effect on CD86 expression compared with the non‐transfected control group.

### 3.2 Overexpression of CD86 enhanced the phagocytosis and killing ability of THP‐1 macrophage

**Figure 2:**
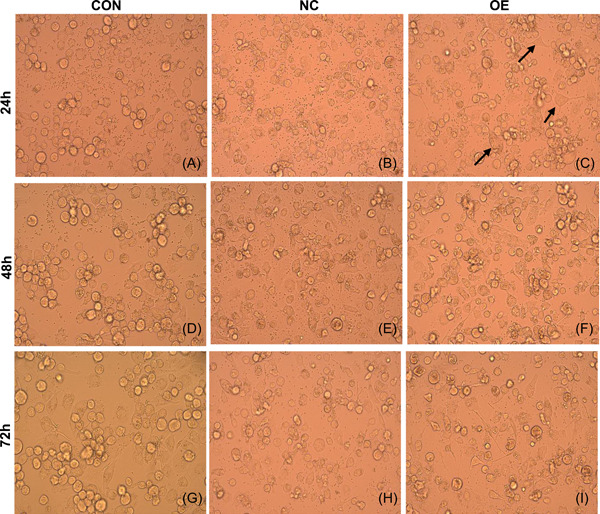
The morphological changes of macrophages during *Talaromyces marneffei* infection were observed under an optical microscope. As early as 24 h OE group had obvious irregular macrophages with long pseudopodia (C), while CON and NC group showed round and oval macrophages (A, B). After 48 h and 72 h *T. marnaffei* infection, the macrophages of OE group still had obvious irregular morphology with long pseudopodia (F, I) in comparison with CON group (D, G) and NC group (E, H). Black arrows indicate irregular macrophages with long pseudopodia. Light microscopy ×400.

**Figure 3:**
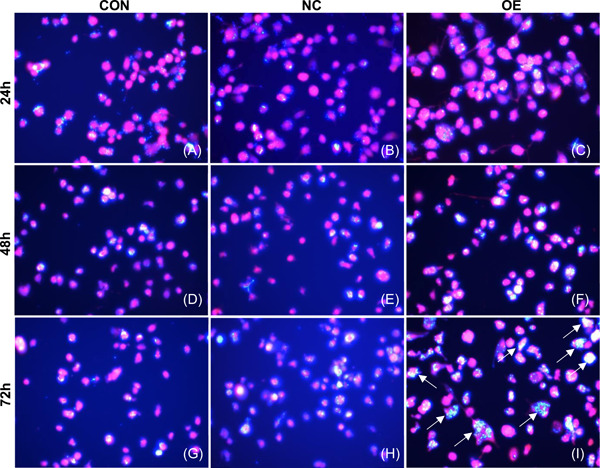
The phagocytosis of macrophages (red) after infection with *Talaromyces marneffei* (bright blue) was observed under fluorescence microscope. (A−F) At 24 and 48 h, there was no significant difference in the number of *T. marneffei* conidia phagocytized by macrophages in CON, NC, and OE group. (G−I) At 72 h, the number of *T. marneffei* conidia phagocytized by macrophages in OE group was significantly higher than that CON and NC group. White arrows indicate macrophages that have engulfed large amounts of *T. marneffei* conidia. Light microscopy ×400.

**Figure 4:**
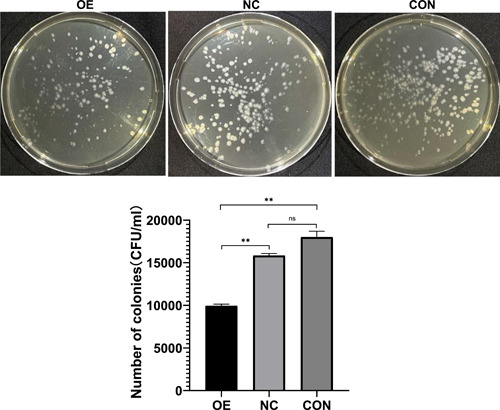
Macrophage killing assay. Colony count of each group = colony count of culture dish × dilution ratio (100 times), compared with CON and NC group, the colony count in OE group decreased significantly. There was no significant difference in colony count between CON and NC group. Data are means ± SD. **p < .01, ^ns^ *p* > .05. SD, standard deviation.

The proportion of irregular macrophages with long pseudopodia was larger and appeared earlier when macrophages were challenged with *T. marneffei* in the OE group in comparison with the CON and NC groups (Figure 2A−I). As early as 24 h, the OE group had obvious irregular macrophages with long pseudopodia (Figure 2C), while the CON and NC groups showed round and oval macrophages (Figure 2A,B). The irregular macrophages with long pseudopodia in the OE group accounted for more than 50% of the cells, while the irregular macrophages in the NC and CON groups accounted for only about 20%. With the passage of infection time, phagocytosis of *T. marneffei* conidia by macrophages gradually increased. At 24 and 48 h, there was no significant difference in the number of *T. marneffei* conidia phagocytized by macrophages in the CON, NC, and OE groups (Figure 3A−F). At 72 h, macrophages in the OE group could still phagocytose more than two times the number of *T. marneffei* conidia compared with the NC and CON groups (Figure 3G−I). In macrophage killing assays, compared with the NC and CON groups, the colony count in the OE group decreased significantly. There was no significant difference in colony count between the CON and NC groups (Figure 4).

### 3.3 Overexpression of CD86 promoted the release of IL‐1β and NO

**Figure 5:**
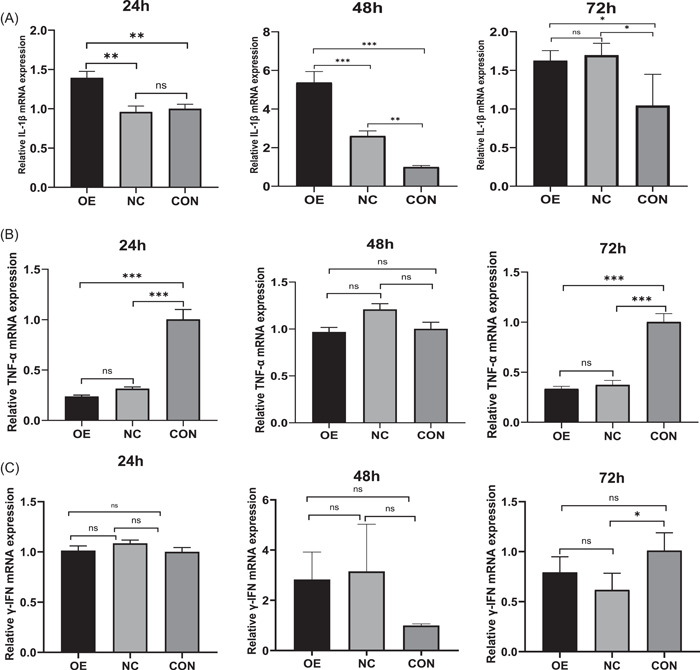
The relative expression of IL‐1β (A), TNF‐α (B), and γ‐IFN (C) mRNA in macrophages. Data are means ± SD. *p < .05, **p < .01, ***p < .001, ^ns^ *p* > .05. SD, standard deviation.

**Figure 6:**
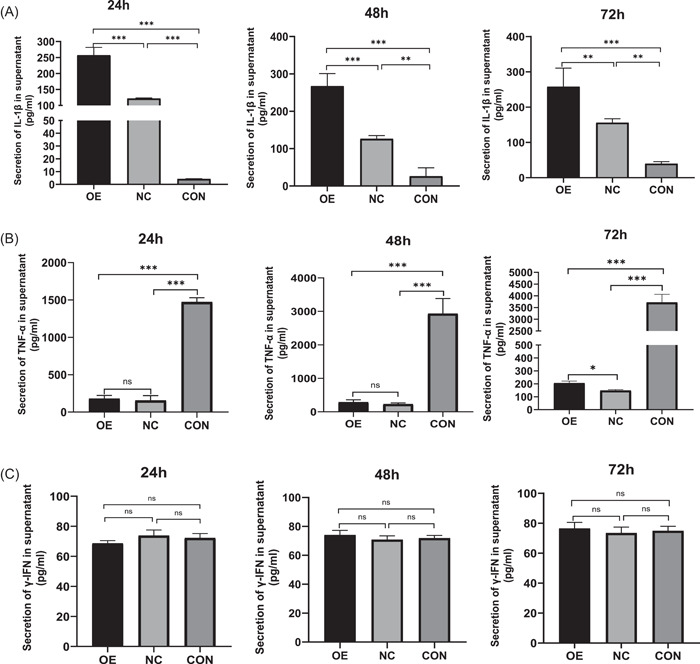
The protein concentration of IL‐1β (A), TNF‐α (B), and γ‐IFN (C) in the supernatant. Data are means ± SD. *p < .05, **p < .01, ***p < .001, ^ns^ *p* > .05. SD, standard deviation.

**Figure 7:**
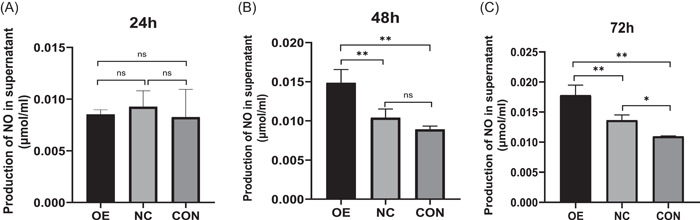
(A−C) NO concentration in the supernatant. Data are means ± SD. *p < .05, **p < .01, ^ns^ *p* > .05. NO, nitric oxide; SD, standard deviation.

When CD86 overexpression was examined for its impact on inflammatory cytokine secretion in *T. marneffei*‐infected macrophages, it was discovered that THP‐1 macrophages that had been stably transfected with Lenti‐CD86 produced more IL‐1β and NO. At 24 and 48 h, relative expression levels of IL‐1β mRNA in macrophages of the OE group were significantly higher than those of the CON and NC groups (Figure 5A). At the same time, IL‐1β in the supernatant of the OE group was significantly higher than that of the CON and NC groups (Figure 6A). It is interesting to note that 24 and 72 h coculture with *T. marneffei*, macrophages from the CON group showed a considerably higher expression of TNF‐α mRNA than those from the OE and NC groups (Figure 5B). TNF‐α levels in the supernatant of CON group were considerably greater at 24 and 48 h than in the OE and NC groups (Figure 6B). Overall, there was no significant difference between the levels of γ‐IFN (Figures 5C and 6C). At 24 h, there was no significant difference in the NO concentration in the supernatant of the three groups (Figure 7A). At 48 and 72 h, the OE group's NO concentration was higher than that of the NC and CON groups' (Figure 7B,C).

## 4 DISCUSSION

Pathogens frequently control the cytokines made by host immune cells during infection to avoid immune responses and aid in their own survival. In vivo evidence for immune evasion in the pathogenesis of *T. marneffei* has already been reported,^2^, ^13^ however the precise pathways are unclear. Monocytes and macrophages engulf *T. marneffei* hiding in the reticuloendothelial system. A surface indicator of M1 macrophage polarization is CD86. The expression of CD86 is increased in the majority of early infection phases. However, compared to cells cultured alone, our earlier research indicated that the expression level of CD86 in macrophages was dramatically reduced, while the quantity of CD86 in the supernatant was significantly increased.^11^, ^12^ An important question that needs to be investigated is the role of CD86 in macrophage infection with *T. marneffei*? Many pattern recognition receptors of macrophages can directly interact with fungi, leading to the production of macrophage inflammatory cytokines. However, one study found that blockade of multiple pattern recognition receptors (CD14, TLR4, tlr1, TLR2, TLR6, and CD18) in the stimulation of human monocytes by the conidia of *T. marneffei* did not cause a reduction in the expression of the cell surface costimulatory molecule CD86.^14^ The CD86 expression of macrophages activated by *T. marneffei* pathogen by a mechanism that is not clearly defined. Research studies have pointed out that *T. marneffei* avoids macrophage killing by modulating the SOCS3‐STAT6 and the TLR9 pathways to induce M2‐like polarization in human THP‐1 macrophages.^15^ Some findings suggest that IL‐6 expression is downregulated during *T. marneffei* infection in bronchial epithelial cells, which may promote *T. marneffei* invasion.^16^ We speculated that *T. marneffei* may adsorb or uptake CD86 in the supernatant produced by macrophages when facing THP‐1 cells to resist the antifungal mechanisms of macrophages. In our study, three groups of macrophages were cocultured with *T. marneffei* in the absence of prestimulated M1 or M2 activation. Lentivirus‐mediated CD86 overexpression in macrophages showed a significant macrophage activation at early stages of infection, while NO and IL‐1β increased significantly showing stronger phagocytosis and killing ability. A study suggested a potential role for the costimulatory receptor CD86/B7‐2 beyond simply promoting competent antigen presentation to T‐cells, but also as a regulator of the anti‐inflammatory IL‐10 response.^17^ It has been stated that IL‐1β upregulates iNOS in osteoarthritis, leading to the rise of NO.^18^ Macrophages can secrete many immunomodulatory agents, such as NO, iNOS, COX‐2, IL‐1β, IL‐6, and TNF‐α.^19^ Pathogens are eliminated by phagocytosis while interactions among these immune regulators occur. Therefore, we speculate that lentivirus mediated CD86 overexpression could activate THP‐1 macrophages by not only enhancing the pinocytic and phagocytic activity but also promoting the production of NO and IL‐1β in THP‐1 cells. We hypothesize that the reduction of CD86 induced by *T. marneffei* results in reduction of antigen presentation by APCs to promote the pathogen invasion.

The ability of pathogenic fungi to switch between multicellular hyphae and unicellular yeast growth forms is a tightly regulated process that requires fungal sensing and response to the host environment, and is critical for pathogenicity.^20^ After inhalation, *T. marneffei* attaches to lung epithelial cells and is subsequently engulfed by lung dendritic cells or macrophages, where it undergoes a phase transition to the yeast phase.^21^ The yeast phase of *T. marneffei* is extremely pathogenic and highly effective at stimulating or manipulating the host immune response.^22^ Massimo et al. found that *T. marneffei* proliferated in yeast‐like cells in unstimulated mouse J774 macrophages after incubation for 24 h. However, when J774 cells were stimulated with gamma interferon and lipopolysaccharide to produce reactive nitrogen intermediates, the proportion of intracellular *T. marneffei* yeast‐like cells was significantly reduced, and the *T. marneffei* conidia were damaged.^23^ However, our research demonstrated that the lentivirus‐mediated overexpression of CD86 in macrophages did not stop *T. marneffei* conidia from developing into yeast cells after being phagocytosed. We hypothesized that *T. marneffei* yeast cells have an ability to bind CD86. We believe that yeast cells of *T. marneffei* coupled to CD86 may increase their pathogenicity. In our study, macrophages that overexpressed CD86 had a greater ability to kill in our macrophage killing assays. We therefore speculate that, in comparison to the control group, CD86 overexpression macrophages may reduce the capacity of *T. marneffei* conidia to convert into yeast cells. However, the number of *T. marneffei* conidia transformed into yeast cells in macrophages cannot be accurately counted, and further experiments are needed to detect the virulence of *T. marneffei* yeast cells. Some studies have suggested that yeast phase specific monoclonal antibody 4D1 (MAb 4D1) can be used as a biomolecular tool to understand phase transition and virulence of *T. marneffei*.^24^ We may further explore the effect of CD86 on *T. marneffei* phase transition by detecting MAb 4D1.

Rong et al. found that macrophage‐derived inflammatory cytokines (TNF‐α, γ‐IFN, IL‐6, IL‐12, IL‐18, IL‐1β) and chemokines (IL‐8, IP‐10) play an important role in the resistance to *T. marneffei* in patients with extreme deficiency of the adaptive immune system after HIV infection. However, inflammatory factors increase dramatically in deceased patients, and an overactive immune response may be associated with disease progression and poor disease outcomes.^25^ Interestingly, we discovered that macrophages transfected with LV‐CD86 or LV‐NC displayed decreased TNF‐α expression compared to the control group. We hypothesized that either CD86 and TNF‐α had no influence on one another, or lentivirus transfection may suppress the production of TNF‐α. In this study we also found that the peak of some proinflammatory cytokines (γ‐IFN and IL‐17A) released mainly by T cells were not as high as those secreted by innate immune cells in patients with extreme deficiency of the adaptive immune system after HIV infection, although γ‐IFN deficiency increased *T. marneffei* infection and proliferation.^25^ In our study, the amount of γ‐IFN detected by the three groups of macrophages was low with no significant difference, which could be related to the absence of T cells. As previously known, the T cell coreceptor CD28/CTL‐A4 interacts with CD80/CD86 in response to the pathogen and triggers a number of signaling pathways, including those regulated by NF‐κB, MAPK, PI3K, and AKT.^26^ The initial reaction to damaging cell stimulation is NF‐κB. Synthesis of cytokines, such as TNF‐α, IL‐1β, IL‐6, and IL‐8, is mediated by NF‐κB.^27^ Uncertainty surrounds the function of CD86 in macrophage activation. Significantly less is known about CD80/CD86 signaling than is known about T cell CD28 signaling. We hypothesized that CD86 overexpression caused by lentiviruses could stimulate macrophage activation by interacting with other immunomodulators, possibly through the MAPK, NF‐κB, and PI3K/AKT signaling pathways.

The study has a number of restrictions. First, THP‐1 macrophage, a single cell line, was employed. The most significant route of transmission is thought to be exposure to bamboo rat feces or inhalation of *T. marneffei* conidia that have been exposed to soil during the rainy season.^28^ Alveolar macrophages would therefore be a better model, but it is challenging to grow stable alveolar macrophages. Currently, the stable alveolar macrophage cells are not commercially available. Moreover, we have not conducted the identical test on a wild‐type cell line. Second, more research must be conducted on the CD86 knockdown cell line and the potential role of CD86 in macrophage resistance to *T. marneffei*. Third, in vitro studies do not replicate the in vivo immunological microenvironment. When an infection occurs in vivo, the immune system, including CD4^+^ T cells, neutrophils, NK cells, and macrophages, work together to coordinate an antifungal response. Last but not least, our study, which is only a pilot study, shows that THP‐1 macrophages more efficiently fight off *T. marneffei* when CD86 is overexpressed. Additional studies are required to better understand the particular mechanism.

## AUTHOR CONTRIBUTIONS

*Conceived and designed the experiments*: Rifeng Chen and Donghua Liu. *Performed the experiments*: Rifeng Chen and Di Yang. *Analyzed the data*: Rifeng Chen, Di Yang, Linxia Shen, and Jinling Fang. *Contributed to the writing of the manuscript*: Rifeng Chen and Raqib Khan.

## CONFLICT OF INTEREST

The authors declare no conflict of interest.
